# The starch-deficient plastidic *PHOSPHOGLUCOMUTASE* mutant of the constitutive crassulacean acid metabolism (CAM) species *Kalanchoë fedtschenkoi* impacts diel regulation and timing of stomatal CO_2_ responsiveness

**DOI:** 10.1093/aob/mcad017

**Published:** 2023-01-20

**Authors:** Natalia Hurtado-Castano, Elliott Atkins, Jerry Barnes, Susanna F Boxall, Louisa V Dever, Jana Kneřová, James Hartwell, John C Cushman, Anne M Borland

**Affiliations:** School of Natural and Environmental Sciences, Newcastle University, Newcastle Upon Tyne NE1 7RU, UK; Plants, Photosynthesis and Soil, School of Biosciences, University of Sheffield, Sheffield S10 2TN, UK; School of Natural and Environmental Sciences, Newcastle University, Newcastle Upon Tyne NE1 7RU, UK; John Innes Centre, Norwich Research Park, Colney Lane, Norwich NR4 7UH, UK; School of Natural and Environmental Sciences, Newcastle University, Newcastle Upon Tyne NE1 7RU, UK; Department of Biochemistry and Systems Biology, Institute of Systems, Molecular and Integrative Biology, University of Liverpool, Liverpool L69 72B, UK; Department of Biochemistry and Systems Biology, Institute of Systems, Molecular and Integrative Biology, University of Liverpool, Liverpool L69 72B, UK; Department of Biochemistry and Systems Biology, Institute of Systems, Molecular and Integrative Biology, University of Liverpool, Liverpool L69 72B, UK; Department of Biochemistry and Systems Biology, Institute of Systems, Molecular and Integrative Biology, University of Liverpool, Liverpool L69 72B, UK; Department of Biochemistry and Molecular Biology, University of Nevada, Reno, NV 89557-0330, USA; School of Natural and Environmental Sciences, Newcastle University, Newcastle Upon Tyne NE1 7RU, UK

**Keywords:** Crassulacean acid metabolism, *Kalanchoë fedtschenkoi*, CO_2_ uptake, stomatal conductance, starch, phosphoglucomutase

## Abstract

**Background and Aims:**

Crassulacean acid metabolism (CAM) is a specialized type of photosynthesis characterized by a diel pattern of stomatal opening at night and closure during the day, which increases water-use efficiency. Starch degradation is a key regulator of CAM, providing phosphoenolpyruvate as a substrate in the mesophyll for nocturnal assimilation of CO_2_. Growing recognition of a key role for starch degradation in C_3_ photosynthesis guard cells for mediating daytime stomatal opening presents the possibility that starch degradation might also impact CAM by regulating the provision of energy and osmolytes to increase guard cell turgor and drive stomatal opening at night. In this study, we tested the hypothesis that the timing of diel starch turnover in CAM guard cells has been reprogrammed during evolution to enable nocturnal stomatal opening and daytime closure.

**Methods:**

Biochemical and genetic characterization of wild-type and starch-deficient RNAi lines of *Kalanchoë fedtschenkoi* with reduced activity of plastidic phosphoglucomutase (PGM) constituted a preliminary approach for the understanding of starch metabolism and its implications for stomatal regulation in CAM plants.

**Key Results:**

Starch deficiency reduced nocturnal net CO_2_ uptake but had negligible impact on nocturnal stomatal opening. In contrast, daytime stomatal closure was reduced in magnitude and duration in the starch-deficient *rPGM* RNAi lines, and their stomata were unable to remain closed in response to elevated concentrations of atmospheric CO_2_ administered during the day. Curtailed daytime stomatal closure was linked to higher soluble sugar contents in the epidermis and mesophyll.

**Conclusions:**

Nocturnal stomatal opening is not reliant upon starch degradation, but starch biosynthesis is an important sink for carbohydrates, ensuring daytime stomatal closure in this CAM species.

## INTRODUCTION

The water-conserving properties of crassulacean acid metabolism (CAM) are mainly a consequence of an inverted diel rhythm of stomatal conductance, which consists of nocturnal opening and closure during the day. CAM is found across 38 families of higher plants worldwide and is thought to have evolved independently multiple times in response to selective pressures forced by water limitation (Yang *et al.*, 2017; Winter *et al.*, 2021). CAM increases water-use efficiency (WUE) by some 6- to 3-fold compared with species that perform C_3_ and C_4_ photosynthesis, respectively, and has been identified as a strategic target for engineering more water-use efficient crops (Borland *et al.*, 2014).

In many CAM species, starch degradation in the leaf mesophyll supports nocturnal CO_2_ uptake through the provision of phosphoenolpyruvate (PEP) as the substrate for the primary fixation step via phosphoenolpyruvate carboxylase (PEPC) during stomatal opening (Borland *et al.*, 2014). This enables the synthesis and nocturnal storage of malate in the vacuole, which is subsequently released and decarboxylated during the day to generate a high internal CO_2_ concentration (*C*_i_), which is believed to maintain stomatal closure during the light period, at least in part (Males and Griffiths, 2017; Lim *et al.*, 2019). Malate also plays a key role within the guard cells of C_3_ plants for osmoregulation and as a counter ion for K^+^ (Santelia and Lawson, 2016). Malate might serve as the main osmolyte responsible for increasing guard cell turgor pressure and stomatal opening at night in CAM plants, with malate being transported from the mesophyll into the guard cells and/or produced directly in the stomatal complex from the degradation of starch within the guard cells (Lee, 2010). Soluble sugars produced by nocturnal starch degradation in the mesophyll and/or within the guard cells could also act as osmolytes or as energy sources to promote stomatal opening (Santelia and Lunn, 2017). Starch degradation clearly plays a central role in regulating the CAM cycle by providing substrate in the mesophyll for assimilation of CO_2_ (Ceusters *et al.*, 2021) but might also have a key role in regulating diel stomatal conductance via the provisioning of energy and osmolytes for the guard cells.

In C_3_ photosynthesis plants, the importance of guard cell starch metabolism for mediating stomatal movements has been demonstrated in *Arabidopsis*, in which starch in the guard cells is broken down almost completely within the first 1 h of the photoperiod, potentially to generate osmolytes and energy required to open stomata at the start of the day (Horrer *et al.*, 2016). Starch degradation in C_3_ guard cells is catalysed by the enzymes β-amylase 1 (BAM1) and α-amylase 3 (AMY3), which are activated by blue light during the first hours of the day, producing malate that acts as a counter ion for K^+^ influx during stomatal opening (Streb and Zeeman, 2012; Santelia and Lunn, 2017). Recently, Abraham *et al.* (2020) reported that diel turnover of starch in the guard cells of the CAM plant *Kalanchoë fedtschenkoi* followed a different pattern from that described for *Arabidopsis*; in particular, the rapid mobilization of starch at the start of the day, which is seen in *Arabidopsis* but was not observed in this CAM species. These observations support the hypothesis that the timing of diel starch turnover in CAM guard cells has been reprogrammed to enable stomatal opening at night and closure during the day. In the present study, we addressed this hypothesis by investigating the importance of starch for nocturnal stomatal opening and daytime closure. Starch-deficient RNAi lines of *K. fedtschenkoi* were generated, in which the plastidic phosphoglucomutase (*PGM*) was silenced using a hairpin RNA encoding binary construct (hereafter referred to as *rPGM* lines). PGM is responsible for the interconversion of glucose 6-phosphate (G6P) into glucose 1-phosphate (G1P), which, in turn, feeds into the pathway of starch synthesis. Plastidic PGM was chosen as the target because it is the most thoroughly defined and characterized starch-deficient mutant of the C_3_ model species *Arabidopsis thaliana* (Caspar *et al.*, 1985). Previously, CAM and starch-deficient mutants of the facultative CAM plant *Mesembryanthemum crystallinum* were found to possess a low activity of plastidic PGM (Cushman *et al.*, 2008*b*). In that work, starch deficiency was shown to impact CAM via substrate limitation of nocturnal C_4_ acid formation, but the consequences of starch deficiency for stomatal regulation over the diel cycle, and for stomatal opening at night in particular, were not considered.

Here, the impact of starch deficiency in *K. fedtschenkoi* on the 24-h cycle of stomatal conductance was assessed using diel gas exchange measurements. In particular, stomatal responses to changes in external [CO_2_] were examined at different points in the diel cycle. The data indicated that stomatal opening at night and at the start of the photoperiod was not reliant upon starch degradation, but daytime stomatal closure was reduced and shortened in the *rPGM* lines. These data are discussed alongside measurements of diel changes in malate and soluble sugar contents in the mesophyll and in guard cell-enriched epidermis, in addition to the transcript abundance of genes implicated in the regulation of guard cell osmolyte balance. Overall, we were able to assess whether the observed changes in stomatal behaviour in the starch-deficient plants were linked to changes in osmolyte metabolism/transport.

## MATERIALS AND METHODS

### *Plant material and generation of transgenic* Kalanchoë fedtschenkoi *lines*

*Kalanchoë fedtschenkoi* ‘Hamet et Perrier’ plants were propagated clonally from the same original accession obtained from the Royal Botanic Gardens, Kew, by Malcolm Wilkins (Wilkins, 1959).

The *rPGM* transgenic lines of *K. fedtschenkoi* were generated by introducing a double-stranded RNA (hairpin RNA) binary construct, designed to target the silencing of the endogenous plastidic *PGM* gene according to the construct design, assembly and plant transformation methods described previously for both *K. fedtschenkoi* and *Kalanchoë laxiflora* (tetraploid) (Dever *et al.*, 2015; Hartwell *et al.*, 2016; Boxall *et al.*, 2017; Boxall *et al.*, 2020). The *PGM* gene family in the diploid *Kalanchoë* genomes decoded to date [namely *K. fedtschenkoi* (two accessions), *K. laxiflora* (two accessions) and *Kalanchoë gracilipes* (one accession)] consists of three members. One plastidic *PGM* (orthologue of JGI Phytozome accession number Kaladp0008s0557.1), characterized by the presence of a chloroplast transit peptide at its N-terminus and through pairwise identity to the *Arabidopsis thaliana* plastidic *PGM* of 82.4 % amino acid level identity, was silenced in *K. fedtschenkoi* using RNAi in this work. In addition, the *Kalanchoë* genomes contain two cytosolic *PGM* isogenes (JGI Phytozome accession numbers Kaladp0057s0141.1 and Kaladp0059s0263.1), which were not targeted by the plastidic *PGM*-specific RNAi construct used here.

Briefly, a 458 bp fragment of the *K. fedtschenkoi PGM* gene (orthologue of JGI Phytozome accession number Kaladp0008s0557.1) was amplified with high-fidelity PCR using KOD Hot-Start DNA polymerase (Merck) and the primers *KfPGM* RNAiF 5ʹ- CACCTCAGAGGTCTTCTTTCACGTTCAGACT-3ʹ and *KfPGM* RNAiR 5ʹ- AAATTTCCAGCCTGTAGGAACCTCATA-3ʹ. The PCR product was TOPO-cloned directionally into the pENTR/D Gateway-compatible entry vector (Life Technologies). The entry clone was recombined using LR Clonase II enzyme mix (Life Technologies) into the Gateway destination vector intron-containing hairpin RNAi binary vector pK7GWIWG2 (II) (Karimi *et al.*, 2002). Constructs were confirmed by DNA sequencing before transformation into *Agrobacterium tumefaciens* strain GV3101. *Agrobacterium*-mediated stable transformation of *K. fedtschenkoi* was achieved using the tissue culture-based method described by Dever *et al.* (2015).

Wild-type and RNAi plants were propagated by the growth of new plantlets on the leaf margin. After 8 weeks, the plantlets were transferred to plastic pots of 127 mm diameter containing John Innes No. 2 compost (J. Arthur Bower’s) and perlite (3:1). Growth conditions were set at 25 °C/19 °C (day/night) and a diurnal photosynthetic photon flux density (PPFD) of 250 µmol m^−2^ s^−1^ at plant height, with a 12 h photoperiod. All measurements were made using CAM-performing leaves (leaf pair 6) of 12-week-old plants. Epidermal tissue and ground mesophyll were harvested separately over a 24 h day/night cycle, using three biological replicates for each time point. *K. fedtschenkoi* is an amphistomatic species; therefore, the epidermal tissue of both abaxial and adaxial leaf surfaces were harvested together as described by Abraham *et al.* (2020). All samples were immediately snap frozen in liquid nitrogen and stored at −80 °C until their evaluation.

### Quantification of titratable acids, starch, soluble sugars and malate

For determining the content of titratable acids, aliquots of methanol extracts were titrated against NaOH (0.05 mm) to a neutral endpoint and expressed as millimoles of H^+^ per gram fresh weight (fwt), as described by Cushman *et al.* (2008*a*). Starch was extracted from the leaf mesophyll as described previously (Haider *et al.*, 2012), and its content in mesophyll was measured as glucose equivalents using the colorimetric phenol/sulphuric acid test described by DuBois *et al.* (1956). To determine starch content in guard cells, the peels were fixed (50 % v/v methanol, 10 % v/v acetic acid) and stained with Lugol’s iodine solution as described by Horrer *et al.* (2016). Soluble sugars were measured in both tissues using the high-pressure ion chromatography (HPIC) technique (Thermo Scientific Dionex), and the amount of sugars (in micromoles per gram fresh weight) was calculated based upon standards of glucose, fructose, sucrose and maltose. Malate content in both mesophyll and guard cell-enriched epidermis was determined by the enzymatic method developed by Hohorst (1970).

### Analysis of gas exchange

Net CO_2_ uptake (in micromoles of CO_2_ per square metre per second), stomatal conductance (in moles of H_2_O per square metre per second) and transpiration (in millimoles of H_2_O per square metre per second) were determined by gas exchange analysis. Three biological replicates of each genotype were evaluated during a 24 h day/night cycle, using the LI-6400XT Portable Photosynthesis System (LI-COR Biosciences). The ambient CO_2_ concentration was set at 400 µmol CO_2_ mol^−1^ air, and the light and temperature were set to track the conditions established in the growth chamber. Integrated instantaneous water-use efficiency (WUEinst) was measured by calculating the area under the curves of net CO_2_ uptake and leaf transpiration at three different times: during the night period, during the light period and over 24 h. To determine stomatal responsiveness to [CO_2_] at night, stomatal conductance was determined under low (50 µmol CO_2_ mol^−1^ air) [CO_2_] administered at different times of the dark period in wild type and *rPGM1a*. Additionally, both wild type and *rPGM1a* were exposed to low (50 µmol CO_2_ mol^−1^ air) and elevated (1600 µmol CO_2_ mol^−1^ air) CO_2_ concentrations at mid-day (phase III), and towards the end of the photoperiod (phase IV). Finally, based on pore length and stomatal density, the maximum theoretical stomatal conductance (*g*_max_) was estimated using the following equation according to Lawson *et al.* (2018):


$$\begin{array}{l} g_{max}=\frac{\frac{Dw}{v}SD.pa_{max}}{pd+\frac{\pi }{2}\sqrt{\frac{pa_{max}}{\pi }}} \end{array}$$

where *Dw* is diffusivity of water vapour in air at 25 °C (0.0000249 m^2^ s^−1^), v is molar volume of air (0.0245 m^3^ mol^−1^), SD is stomatal density (stomata per square metre), $pa_{max}$is maximum stomatal pore area (in square metres) calculated as an ellipse, pd is stomatal pore depth (in metres) considered to be equivalent to the width of a turgid guard cell, and $\frac{\pi }{2}\sqrt{\frac{pa_{max}}{\pi }}$ is the ‘end correction’ that takes into account the influence of diffusion shells from outside the end of the stomatal pore (Lawson *et al.*, 2018).

### Transcript abundance of genes using real-time qPCR

Real-time qPCR was used to confirm silencing of Kf-*pgm1* and to investigate the transcript abundance of several genes encoding enzymes involved in starch, sugar and malate metabolism and which have been implicated in stomatal regulation. These genes included the ATP-binding cassette malate transporter (Kf-*ABCB14*), the sucrose synthase 1 (Kf-*SUSY1*) and the sugar transporter (Kf-*STP1*). RT-qPCR measurements were made to assess differences in abundance and day/night timing of maximal gene expression in guard cell-enriched epidermis and mesophyll cells in *rPGM1a* and wild type. To determine the relative quantity, the 2^−ΔΔ*Ct*^ method (Livak and Schmittgen, 2001) was performed, normalizing the data against the cDNA calibrator and the *K. fedtschenkoi* thioesterase/thiol ester dehydrase-isomerase (Kf-TEDI) superfamily protein (Phytozome Kaladp0068s0118.1), as reported by Boxall *et al.* (2017). In addition, no-template controls (NTC) were added to confirm the absence of contamination.

### Statistical analysis

To determine significant differences, an ANOVA and LSD (least significant difference) *post hoc* test were performed. The data obtained from gas exchange experiments were analysed with the non-parametric Mann–Whitney *U*-test, based upon its non-normality and the non-homogeneity determined with the Shapiro–Wilk test and Levene’s test, respectively. All the statistical analyses were done using SPSS software (IBM Corp. Released 2016. IBM SPSS Statistics for Windows, Version 24.0; IBM, Armonk, NY, USA).

## RESULTS

### *Selecting a starch-deficient line of* K. fedtschenkoi

**Fig. 1.**
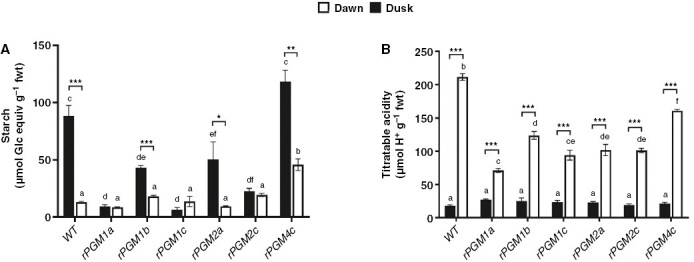
Leaf starch content (in micromoles of glucose equivalent per gram fresh weight; A) and leaf titratable acidity (in micromoles of H^+^ per gram fresh weight; B) in wild type and six independent RNAi lines (*rPGM1a*, *rPGM1b*, *rPGM1c*, *rPGM2a, rPGM2c* and *rPGM4c*) of *Kalanchoë fedtschenkoi*. Leaves were sampled at the start (dawn; white columns) and end (dusk; black columns) of the photoperiod. The error bars indicate the s.e. of three biological replicates. Different letters indicate significant statistical difference among lines determined by one-way ANOVA with Tukey’s post hoc test (*P* ≤ 0.05). *Significant statistical difference between time points determined by one-way ANOVA (*P ≤* 0.05). **Significant statistical difference between time points determined by one-way ANOVA (*P ≤* 0.01). ***Significant statistical difference between time points determined by one-way ANOVA (*P ≤* 0.001).

**Fig. 2.**
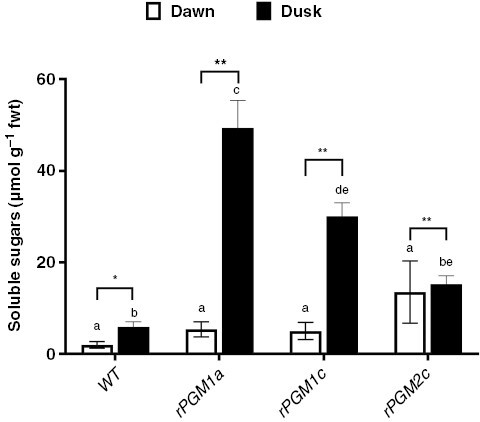
Leaf soluble sugars content (in micromoles per gram fresh weight) in wild type and in three independent RNAi lines (*rPGM1a*, *rPGM1c* and *rPGM2c*) of *Kalanchoë fedtschenkoi*. Leaf pair 6 was used for this analysis. Leaves were sampled at the start (dawn; white columns) and end (dusk; black columns) of the photoperiod. The error bars indicate the s.e. of three biological replicates. Different letters indicate significant statistical difference among lines determined by one-way ANOVA with Tukey’s post hoc test (*P* ≤ 0.05). *Significant statistical difference between time points determined by one-way ANOVA (*P* ≤ 0.05). **Significant statistical difference between time points determined by one-way ANOVA (*P* ≤ 0.01).

**Fig. 3.**
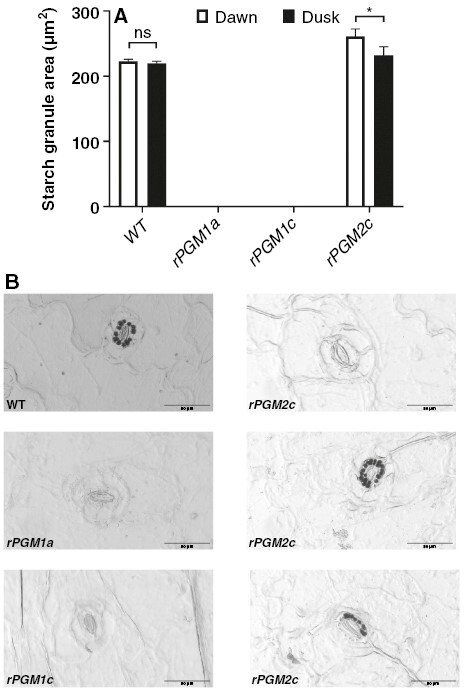
(A) Starch granule area (in square micrometres) in guard cells (upper and lower epidermal surface) of wild type and in three independent RNAi lines (*rPGM1a*, *rPGM1c* and *rPGM2c*) of *Kalanchoë fedtschenkoi*. Samples were taken at the start of the photoperiod. The bars indicate the s.e. of 80 replicates (four biological replicates, each with 20 views per replicate). *Significant statistical difference between time points determined by one-way ANOVA (*P* < 0.05); ns, not significant. (B) Starch deposits in guard cells of wild type and *PGM* RNAi lines of *K. fedtschenkoi*. *rPGM1a* and *rPGM1c* lack starch in the guard cells, whereas *rPGM2c* presents variations in starch content (starch similar to wild type, no starch and presence in a single guard cell). The tissue corresponds to epidermal peels from leaf pair 6. Scale bars represent 50 µm.

**Fig. 4.**
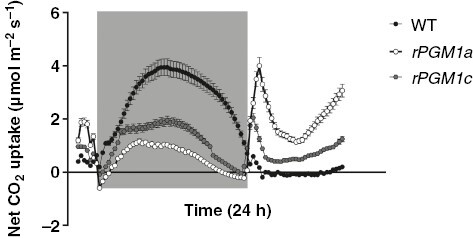
Net CO_2_ uptake of wild-type (black symbols), *rPGM1a* (white symbols) and *rPGM1c* (grey symbols) lines of *Kalanchoë fedtschenkoi*, during a 24 h period (grey background indicates night period). The error bars represent the s.e. of three replicates.

Our first aim was to isolate a starch-deficient RNAi line of *K. fedtschenkoi* that could be used to examine how a lack of starch impacted on gas exchange characteristics and stomatal responses to [CO_2_]. Six independent RNAi lines for which the single copy of the plastidic *PGM* gene had been targeted for silencing with a hairpin RNA transgene were tested for leaf starch content and titratable acids measured at the start (dawn) and end (dusk) of the photoperiod. Lines *rPGM1a*, *rPGM1c* and *rPGM2c* were found to have the lowest leaf starch contents at both dawn and dusk, and the overnight accumulation of titratable acids in these lines was also reduced compared with wild type (Fig. 1). Consequently, *rPGM1a*, *rPGM1c* and *rPGM2c* were evaluated further in order to select the best line for this study. Accumulation of soluble sugars, which is a phenotype consistent with the starch deficiency resulting from the loss of plastidic PGM activity in the *Arabidopsis thaliana* mutant (Caspar *et al.*, 1985; Hanson and McHale, 1988), was measured in these three RNAi lines and compared with wild type. Of the three RNAi lines analysed, line *rPGM1a* showed the most significant accumulation of soluble sugars measured at the end of the photoperiod (Fig. 2). Both *rPGM1a* and *rPGM1c* also showed a complete lack of starch in the guard cells, whereas *rPGM2c* showed variations in the starch content of guard cells (Fig. 3). Lines *rPGM1a* and *rPGM1c* were subsequently compared with wild type in terms of diel patterns of leaf gas exchange. Both lines showed lower rates of nocturnal net CO_2_ uptake compared with wild type, whilst daytime net CO_2_ uptake was substantially higher in the RNAi lines compared with wild type, where the absence of net CO_2_ uptake for most of the light period indicated complete stomatal closure (Fig. 4). Of these two RNAi lines, *rPGM1a* showed the largest reduction in nocturnal net CO_2_ uptake, combined with elevated light period net CO_2_ uptake relative to wild type, and was thus taken forward for further analyses.

**Fig. 5.**
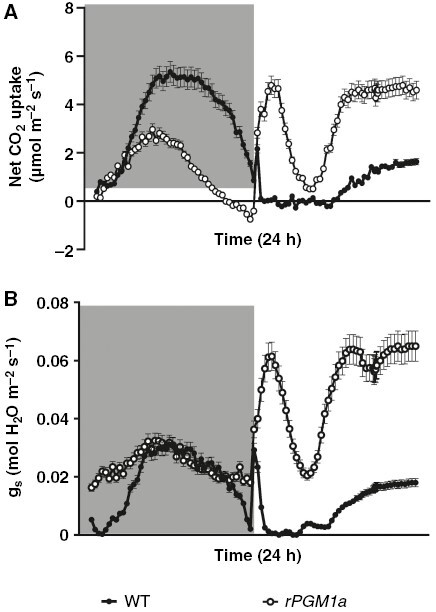
Net CO_2_ uptake (A) and stomatal conductance (B) of wild-type (black symbols) and *rPGM1a* (white symbols) plants of *Kalanchoë fedtschenkoi*, during 24 h (grey background indicates night period). The error bars represent the s.e. of three replicates.

In *rPGM1a*, the lower net CO_2_ uptake during the night and the unaffected stomatal conductance compared with wild type (Fig. 5A) suggest that the lower CAM activity of *rPGM1a* was attributable to a deficiency in PEP carboxylation, probably because of a lack of substrates and not owing to reduced stomatal opening. In addition, during the day period, the *rPGM1a* line was not able to close the stomata like the wild type (Fig. 5B). The integrated CO_2_ uptake and leaf transpiration over a 24 h period indicated that instantaneous WUEinst was significantly higher (*P* ≤ 0.05) in wild type compared with *rPGM1a* during the light period but not at night (Table 1). Additionally, anatomical measurements of stomatal size and density (Supplementary data Fig. S1) showed that the maximum theoretical stomatal conductance (*g*_max_) was significantly higher (*P* ≤ 0.05) in *rPGM1a* compared with wild type (451.383 ± 9.384 and 355.823 ± 15.429 mmol m^−2^ s^−1^, respectively), implying that starch deficiency conferred an anatomical predisposition towards potentially higher stomatal conductance.

**Table 1.** Instantaneous water-use efficiency (WUEinst; in micromoles of CO_2_ per mole of H_2_O) for wild type and *rPGM1a*. The WUEinst was calculated as the ratio of integrated net CO_2_ uptake (in micromoles of CO_2_ per square metre) to integrated net water loss (in moles of H_2_O per square metre) over the entire 24 h cycle. The data correspond to the mean and s.e. of three biological replicates

Genotype	Time period	Integrated CO_2_ uptake (µmol CO_2_ m^−2^)	WUEinst (µmol CO_2_ mol^−1^ H_2_O)
*rPGM1a*	Night	57.048 ± 10.537	9.705 ± 2.72
	24 h	72.821 ± 12.206	5.896 ± 1.894
	Day	6.830 ± 1.319	6.080 ± 1.350
Wild type	Night	109.352 ± 4.699	12.706 ± 0.429
	24 h	104.689 ± 2.436	8.708 ± 0.871
	Day	2.878 ± 1.337	7.057 ± 2.818

### Diel changes in starch, malate and soluble sugars in leaf mesophyll and guard cell-enriched epidermis

**Fig. 6.**
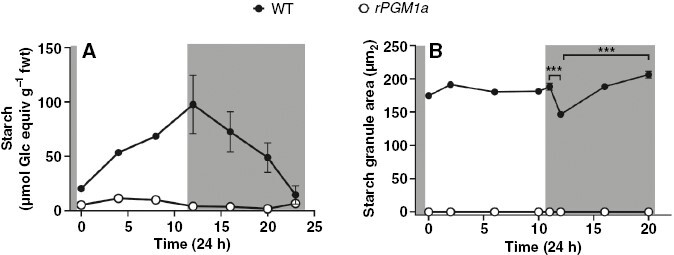
Starch content in wild-type (black symbols) and *rPGM1a* (white symbols) plants of *Kalanchoë fedtschenkoi*, during 24 h (grey background indicates night period). (A) Total leaf starch (in micromoles of glucose equivalent per gram fresh weight) was quantified by colorimetric assay; the error bars indicate the s.e. of three biological replicates. (B) Starch granule area (in square micrometres) in guard cells was averaged between abaxial and adaxial epidermal surfaces; the error bars indicate the s.e. of 120 replicates (three biological replicates, each with 40 views per replicate). ***Significant statistical difference between time points determined by one-way ANOVA (*P* ≤ 0.001).

In the wild type, starch content increased in the mesophyll over the course of the photoperiod and was steadily and almost completely depleted during the night (Fig. 6). Starch content was negligible in mesophyll of *rPGM1a*, confirming the starch-deficient phenotype of this line, as indicated by the initial screen described above (Figs 1A and 6A). The guard cell starch content in the wild type, represented as starch granule area (in square micrometres), showed no significant change in content over the course of the light period. There was significant (*P* ≤ 0.05) net depletion of guard cell starch during the first 1 h of darkness, but thereafter the guard cell starch increased gradually over the course of the night (Fig. 6B).

**Fig. 7.**
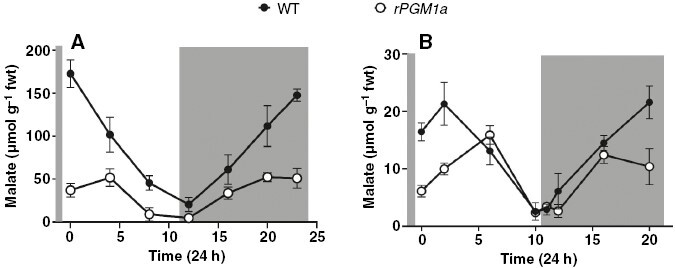
Malate content (in micromoles per gram fresh weight) in mesophyll (A) and guard cell-enriched epidermis (B) of wild-type (black symbols) and *rPGM1a* (white symbols) plants of *Kalanchoë fedtschenkoi*, during 24 h (grey background indicates night period). Error bars indicate the s.e. of six replicates (three biological replicates, each with two technical replicates).

Diel changes in malate content in both genotypes represented the typical CAM turnover of this organic acid, with diurnal degradation owing to decarboxylation and nocturnal accumulation as a product of PEP carboxylation by PEPC (Fig. 7). Diel turnover of malate in the mesophyll was significantly higher (3.5-fold higher) in wild type compared with *rPGM1a* (Fig. 7A). Nocturnal accumulation and daytime mobilization of malic acid were also evident in the guard cell-enriched epidermis (Fig. 7B). However, on a fresh weight basis, diel turnover of malate in wild type was more than eight times higher in the mesophyll cells compared with the epidermal peels. In the epidermis of *rPGM1a*, malate accumulated over the first half of the night, in line with changes in malate content in the wild-type epidermis. However, over the second half of the night, malate content continued to increase in the wild-type epidermis, but no further net increase in malate content was observed in the epidermis of *rPGM1a*. The most marked differences in diel changes in epidermal malate content were observed during the photoperiod (Fig. 7B). In wild-type epidermis, malate content declined over the 12 h light period, whereas in *rPGM1a*, malate content increased over the first half of the photoperiod, peaking in the middle of the day, before decreasing over the remaining 6 h of the photoperiod.

**Fig. 8.**
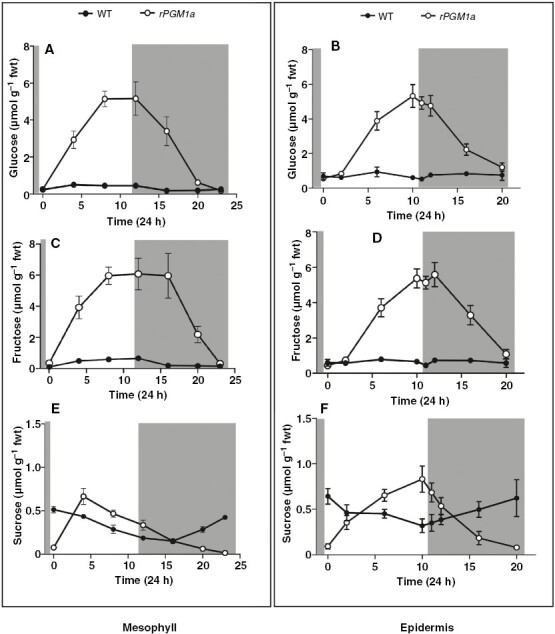
Glucose (A, B), fructose (C, D) and sucrose (E, F) content (in micromoles per gram fresh weight) in mesophyll (left panels) and guard cell-enriched epidermis (right panels) of wild-type (black symbols) and *rPGM1a* (white symbols) plants of *Kalanchoë fedtschenkoi*, during 24 h (grey background indicates night period). Error bars indicate the s.e. of six replicates (three biological replicates, each with two technical replicates).

Diel soluble sugar content was significantly different (*P* ≤ 0.05) between wild type and *rPGM1a*. Glucose and fructose contents in both mesophyll and epidermis increased during the day in *rPGM1a*, reaching maximum values at the end of the photoperiod that were ~7-fold higher than those in wild type (Fig. 8A–D). Sucrose was present in lower amounts compared with glucose and fructose, and although sucrose contents were comparable between wild type and *rPGM1a*, there were marked differences between genotypes and between mesophyll and epidermis in terms of diel turnover. In wild type, sucrose was depleted during the day and then accumulated over the subsequent night in both mesophyll and epidermis. In *rPGM1a*, sucrose accumulated in the mesophyll over the first few hours of the photoperiod and was subsequently depleted over the remainder of the photoperiod and over the night, whereas sucrose in the epidermis increased steadily over the photoperiod and was subsequently degraded during the following night (Fig. 8E, F).

Stoichiometric analysis was performed to determine the relationship between nocturnal degradation of carbohydrates and malate accumulation during phase I in both the mesophyll and guard cell-enriched epidermis and to compare the starchless phenotype of *rPGM1a* and the lack of significant starch degradation in wild-type guard cells. Starch, soluble sugars and malate were quantified at dawn and dusk (Table 2). The breakdown of mesophyll starch is measured as glucose equivalents, where each mole of glucose potentially produces two moles of PEP via glycolysis, and each mole of PEP is carboxylated to produce one mole of malate. PEP excess or deficit was calculated as the difference between the amount of PEP required for nocturnal malate accumulation minus the PEP produced from glycolytic breakdown of all the different carbohydrate sources. The calculations suggested that nocturnal malate formation in wild-type mesophyll was derived from breakdown of starch and hexoses (PEP excess of 3.036 µmol). Regarding *rPGM1a*, the higher accumulation of soluble sugars in the mesophyll was probably responsible for nocturnal PEP formation by glycolysis, as a compensation for the absence of starch. However, the lower malate turnover overall in *rPGM1a* together with the deficit in PEP (20.230 µmoles) indicate the importance of starch turnover for CAM activity. Given that starch in guard cell-enriched epidermis was measured as starch granule area, it was not possible to calculate the relationship with PEP turnover in this tissue.

**Table 2.** Nocturnal malate accumulation from breakdown of carbohydrates in mesophyll and guard cell-enriched epidermis of wild-type and *rPGM1a* plants, based on stoichiometry calculations. The phosphoenolpyruvate (PEP) excess or deficit was calculated as the difference between the amount of PEP required for nocturnal malate accumulation minus the PEP produced from glycolytic breakdown of all the different carbohydrate sources (NC refers to not calculated)

Tissue	Genotype	PEP available from starch (µmol g^−1^ fwt)	PEP available from sucrose (µmol g^−1^ fwt)	PEP available from glucose (µmol g^−1^ fwt)	PEP available from fructose (µmol g−^1^ fwt)	Malate accumulation (µmol g^−1^ fwt)	Excess/deficit PEP
Mesophyll	Wild type	154.493	−0.656	0.43	1.106	152.337	3.036
	*rPGM1a*	−2.62	0.257	4.908	11.468	34.244	−20.23
Epidermis	Wild type	NC	−0.291	−0.334	−0.147	13.53	−14.302
	*rPGM1a*	NC	0.592	8.715	4.683	2.627	11.363

### Impact of starch deficiency on leaf gas exchange and stomatal responses to [CO_2_]

**Fig. 9.**
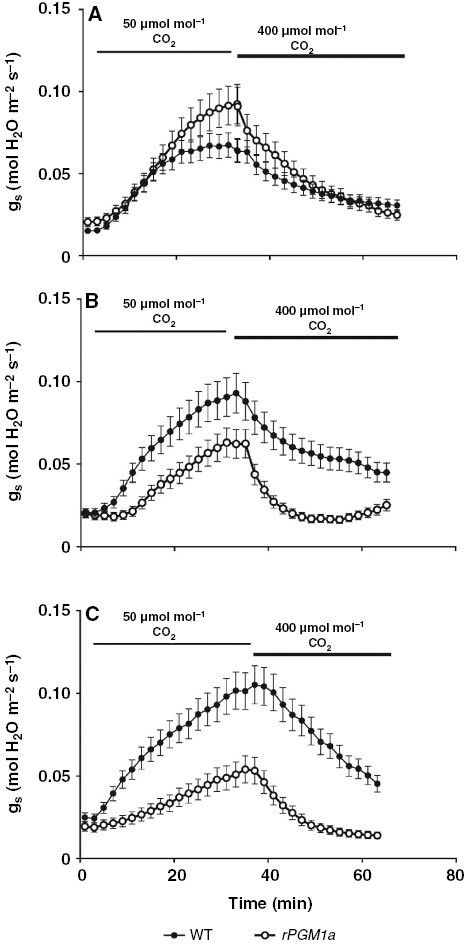
Responses of stomatal conductance (*g*_s_) in wild-type (black symbols) and *rPGM1a* (white symbols) plants of *Kalanchoë fedtschenkoi* to altered [CO_2_] (50 or 400 μmol mol^−1^ CO_2_) imposed at different times from start of the dark period [(A) early, 40 min into dark period; (B) mid, 4.5 h into dark period; (C) late, 8.2 h into dark period)]. The error bars represent the s.e. of three replicates.

In order to establish whether starch deficiency affects stomatal responsiveness to [CO_2_] at night, we compared stomatal responses to low (50 µmol CO_2_ mol^−1^ air) [CO_2_] administered at different times of the dark period in wild type and *rPGM1a* (Fig. 9). The data indicate that although starch deficiency did not, in general, curtail stomatal opening in response to low [CO_2_], the responsiveness of stomatal conductance (i.e. the speed and magnitude of opening) to low [CO_2_] differed between genotypes at different points in the night. Thus, stomatal responsiveness to low [CO_2_] increased as the night progressed in wild type, but the opposite was seen in *rPGM1a*, where stomatal responsiveness to low [CO_2_] declined over the course of the night (Fig. 9).

**Fig. 10.**
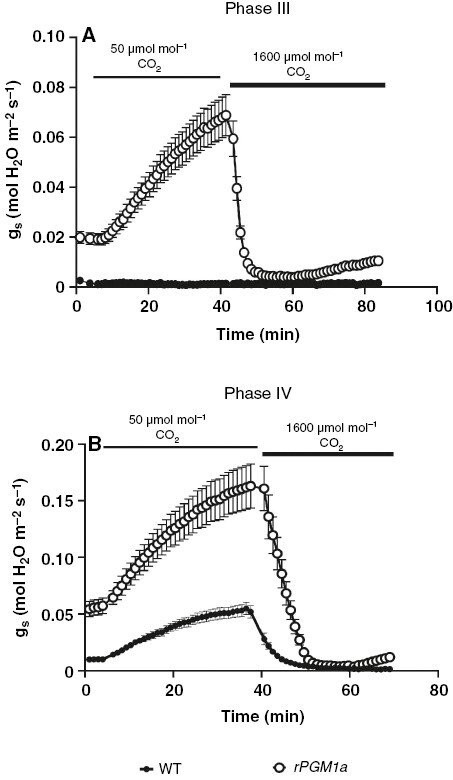
Stomatal conductance (*g*_s_) of wild-type (black symbols) and *rPGM1a* (white symbols) plants of *Kalanchoë fedtschenkoi*, during exposure to 50 and 1600 μmol mol^−1^ of CO_2_ during a 24 h period of CAM phases III (A) and IV (B). The error bars represent the s.e. of three replicates.

In terms of stomatal behaviour during the photoperiod, reduced nocturnal malate accumulation in *rPGM1a* might consequently result in a lower internal *C*_i_ during malate decarboxylation, such that the *C*_i_ was insufficient to mediate stomatal closure. Thus, we predicted that exposing leaves of the starch-deficient line to high [CO_2_] during the day would result in complete closure of stomata. Both wild-type and *rPGM1a* plants were exposed to low (50 µmol CO_2_ mol^−1^ air) and elevated (1600 µmol CO_2_ mol^−1^ air) CO_2_ concentrations at mid-day (phase III), and towards the end of the photoperiod (phase IV), in order to determine whether starch deficiency impacted the daytime stomatal response to [CO_2_]. Data revealed that in phase III, when wild-type stomata were closed, changes in the external atmospheric CO_2_ concentration did not have any effect on stomatal conductance (Fig. 10A). In *rPGM1a*, exposure to low [CO_2_] in phase III resulted in a sustained increase in stomatal conductance, followed by stomatal closure within 11 min when plants were exposed to elevated [CO_2_]. However, stomata of *rPGM1a* did not remain closed under elevated CO_2_ and partly re-opened after 16 min (Fig. 10A). Later in the photoperiod during phase IV, both wild type and *rPGM1a* opened stomata in response to low [CO_2_], with the speed and magnitude of opening being more pronounced in the starch-deficient plants. Exposure to high [CO_2_] resulted in stomatal closure in both genotypes. However, in *rPGM1a*, an inability to maintain complete stomatal closure under elevated [CO_2_] was again observed, as stomata started to re-open after 14 min under elevated [CO_2_] (Fig. 10B).

**Fig. 11.**
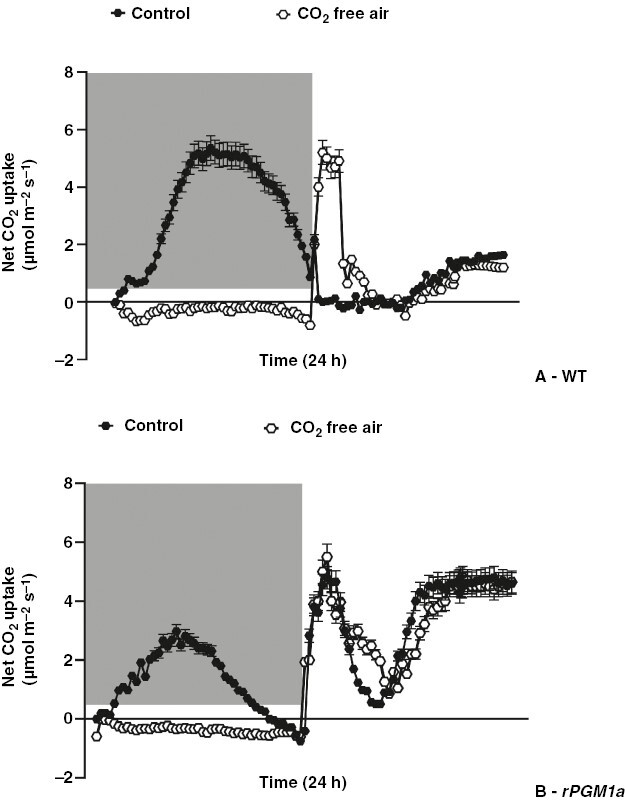
Net CO_2_ uptake of wild-type (A) and *rPGM1a* (B) plants of *Kalanchoë fedtschenkoi* monitored over 24 h under 400 µmol CO_2_ mol^−1^ (black symbols) or under CO_2_-free air treatment at night (white symbols). The grey background indicates the night period. The error bars represent the s.e. of three replicates.

As an additional way of testing how stomata of wild type and *rPGM1a* responded to altered daytime *C*_i_, plants were exposed to CO_2_-free air during the night to curtail nocturnal malate synthesis and then released into ambient [CO_2_] (i.e. 400 µmol CO_2_ mol^−1^ air) at the start of the photoperiod (Fig. 11). The gas exchange profiles obtained were compared with those measured in control conditions of ambient [CO_2_] given during the night and photoperiod. Data showed that after a night in CO_2_-free air, both genotypes fixed CO_2_ during the first hours of the day, and this was followed by a reduction in net CO_2_ uptake, indicating stomatal closure. Importantly, the data indicate that curtailing nocturnal malate accumulation delayed, but did not prevent, daytime closure of stomata in wild type (Fig. 11A). In the starch-deficient *rPGM1a*, curtailing nocturnal malate accumulation by a night in CO_2_-free air had a negligible impact on subsequent daytime gas exchange (Fig. 11B). Together, these experiments suggest that factors other than changes in *C*_i_ regulate diurnal stomatal behaviour in CAM plants and that starch metabolism has important implications for daytime stomatal regulation.

### Transcript abundance of genes related to stomatal regulation

A significant reduction in transcript abundance of Kf*-PGM1* in both mesophyll and guard cell-enriched epidermis of *rPGM1a* sampled at dawn and dusk confirmed the RNAi-directed downregulation of this gene. In wild type, Kf*-PGM1*transcript levels were significantly higher in the mesophyll compared with the epidermis (Supplementary data Fig. S2A). Regarding malate active transport, the ATP-binding cassette transporter *ABCB14*, for which the encoded protein is located in the guard cell plasma membrane, mediates malate uptake from the apoplast in *Arabidopsis thaliana* (Lee *et al.*, 2008). In *K. fedtschenkoi*, transcript abundance of Kf*-ABCB14* was highest in epidermal peels of the wild type at the beginning of the night, whereas in *rPGM1a* this transcript was most abundant at the beginning of the day (Supplementary data Fig. S2B). Given the higher diurnal accumulation of sugars in *rPGM1a*, the transcript abundance of the sugar transporter protein 1 (*STP1*) was also evaluated in both genotypes. In *Arabidopsis thaliana*, this transporter, located in the plasma membrane of guard cells, is involved in the import of monosaccharides to sustain osmoregulation on stomatal opening (Stadler *et al.*, 2003). The highest transcript abundance of Kf*-STP1* was noted at dawn in the guard cell-enriched epidermis of *rPGM1a*, where the level of this transcript was significantly higher than that in wild type (Supplementary data Fig. S2C). If activity follows transcript abundance, the data imply enhanced import of sugars to guard cells of the starch-deficient plants at the start of the photoperiod. Additionally, given the sucrolytic activity of sucrose synthases (*SUSY*) in the conversion of sucrose into fructose and UDP-glucose (UDPG), and the importance of sucrose turnover on stomatal metabolism, the regulation of Kf*-SUSY1* and Kf-*SUSY3* was also determined in both lines. No significant difference in Kf-*SUSY3* expression was found between genotypes (data not shown). The peak in Kf*-SUSY1* transcript was restricted to the beginning of the photoperiod, with differences between genotypes. In wild type, transcript abundance of Kf*-SUSY1* was higher in the mesophyll (Supplementary data Fig. S2D). In contrast, in *rPGM1a* this transcript was highly abundant in the guard cell-enriched epidermis, suggesting that guard cells of *rPGM1a* have a unique metabolic machinery compared with mesophyll and/or that regulation of sucrose metabolism differs between mesophyll and epidermis in the absence of significant plastidic PGM activity.

## DISCUSSION

### Nocturnal stomatal opening is not reliant on starch degradation

Starch degradation plays a central role in regulating the CAM cycle by providing the sugars that are metabolized through glycolysis to PEP, the substrate for PEPC in the mesophyll to assimilate atmospheric CO_2_ in the dark period (Ceusters *et al.*, 2021). The aim of the present study was to assess whether starch degradation could also have a key role in CAM by being important for the correct regulation of nocturnal stomatal opening via the provisioning of energy and osmolytes to increase guard cell turgor at night. Previous work using the facultative CAM species *M. crystallinum* indicated that mutants deficient in phosphoglucomutase (PGM) were CAM and starch deficient (Cushman *et al.*, 2008*a*; Haider *et al.*, 2012), but the impact on stomatal behaviour was not assessed. To address this question, we used RNAi to generate PGM-deficient transgenic lines of the model constitutive CAM species, *K. fedtschenkoi*. We screened six independent RNAi lines for CAM activity and starch content and found two independent lines (*rPGM1a* and *rPGM1c*) that lacked starch in the leaf ground mesophyll and guard cells and that showed reduced nocturnal net CO_2_ uptake and malate accumulation relative to the wild type. Line *rPGM1a* had the most extreme phenotype in terms of starch deficiency and reduced CAM activity, but nocturnal stomatal conductance was comparable to that of wild type. These data support the view that reduced CAM in lines with reduced plastidic *PGM* activity, which leads to starch-deficient plants, is attributable to a limitation in the nocturnal supply of sugars for PEP synthesis, rather than a consequence of diffusional limitation to atmospheric CO_2_ uptake into the leaves for nocturnal carboxylation (Cushman *et al.*, 2008*a*; Borland *et al.*, 2016). Thus, the production of ATP and osmolytes required for nocturnal stomatal opening in *K. fedtschenkoi* is not reliant on starch degradation in the guard cells or mesophyll cells.

### Impact of starch deficiency on nocturnal osmolyte balance in guard cell-enriched epidermis

Malate is generally accepted as the predominant anion during stomatal opening and closing in C_3_ photosynthesis species (Fernie and Martinoia, 2009) and has also been proposed as the main osmolyte responsible for nocturnal stomatal opening in CAM plants (Lee, 2010). Our data support this hypothesis, with nocturnal accumulation of malate measured in guard cell-enriched epidermis of both wild-type and starch-deficient *K. fedtschenkoi*. Furthermore, the accumulation of malate was severalfold higher (on a molar basis) compared with that of soluble sugars. The source of this guard cell malate is still a matter of debate. Previous proteomics analysis of guard cell-enriched epidermis of *K. fedtschenkoi* indicated the presence of phosphorylated (active) PEPC at night (Abraham *et al.*, 2020), indicating that malate could potentially be produced directly in the stomatal complex using PEP generated from the degradation of guard cell starch and/or sugars. Clearly, malate accumulation in the epidermal tissue of *rPGM1a* did not require starch degradation, and there was limited net breakdown of guard cell starch over the course of the night in wild-type *K. fedtschenkoi*, although malate content increased steadily in the guard cell-enriched epidermis throughout the night. Glycolytic processing of soluble sugars in the epidermis to provide sufficient PEP for night-time synthesis of malate via PEPC appeared to be stoichiometrically feasible in the starch-deficient *rPGM1a* but not in wild type (Table 2). Such findings suggest that in wild-type *K. fedtschenkoi*, malate was transported from underlying mesophyll cells into the guard cells, rather than synthesized *in situ*.

In *Arabidopsis*, the ATP-binding cassette transporter ABCB14, which is located in the guard cell plasma membrane, mediates malate uptake from the apoplast and also coordinates the response of stomata to changes in internal CO_2_ concentration (Lee *et al.*, 2008). Transcript abundance of the *K. fedtschenkoi ABCB14* orthologue was significantly enriched in wild-type epidermis at the start of the night, which, if transcript abundance coordinates transporter activity, would be consistent with the hypothesis that nocturnal import of malate to guard cells promotes stomatal opening at night. Accumulation of malate in the mesophyll and its transport to guard cells via ABCB14 could be an important hub that connects CAM photosynthesis in the mesophyll with stomatal behaviour over the diel cycle. A contrasting scenario was revealed for the starch-deficient *rPGM1a* plants, in which Kf-*ABCB14* transcripts were barely detected at night, but instead increased in abundance at the start of the photoperiod. This was coincident with the sustained net accumulation of malate in guard cell-enriched epidermis of the mutant line over the first half of the light period, at a time when malate was declining in wild-type epidermis (Fig. 7). The earlier suggestion that malate could be synthesized directly within the guard cells of the *rPGM1a* plants using soluble sugars to provide a substrate for nocturnal anapleurotic CO_2_ fixation, presents the hypothesis that night-time import of malate to guard cells in the starch-deficient line is quantitatively less important than in wild type. Ultimately, genetic manipulation of Kf-*ABCB14* in *K. fedtschenkoi* using RNAi and/or CRISPR-Cas9-mediated mutagenesis (Liu *et al.*, 2019) will be necessary to establish whether this gene plays a similar role to the *Arabidopsis* orthologue in terms of mediating malate import to the *K. fedtschenkoi* guard cells.

### Starch deficiency impacts the timing of stomatal responsiveness to [CO_2_] over the night

A key trigger for nocturnal opening of stomata in CAM is believed to be driven by reduced *C*_i_ when PEPC activity increases at dusk (von Caemmerer and Griffiths, 2009; Males and Griffiths, 2017). Stored products in the guard cells and signals from the mesophyll are believed to influence stomatal response to changes in *C*_i_ in *K. fedtschenkoi* (Santos *et al.*, 2021). We hypothesized that starch deficiency and the resultant changes in sugar and malate homeostasis in both mesophyll and guard cells would impact on nocturnal responses to [CO_2_] in this CAM species. Our data indicated that starch degradation was not required to facilitate stomatal opening in response to low external [CO_2_] administered at night, and the magnitude and speed of this response was not reliant on starch degradation per se. However, although stomatal responsiveness (i.e. the speed and magnitude of opening) to low external [CO_2_] increased over the course of the dark period in wild type, in the starch-deficient plants stomata became less responsive to low external [CO_2_] as the night progressed. Circadian gating of stomatal responsiveness to CO_2_ has been discussed previously within the context of CAM (Borland *et al.*, 2014; Hartwell, 2006). Stomatal responses to [CO_2_] in *K. fedtschenkoi* appear to be influenced by the presence of the mesophyll, but are not mediated solely through changes in *C*_i_ (although these clearly play an important role; Santos *et al.*, 2021). In CAM, it appears that some other unknown diffusible mesophyll signal coordinates stomatal behaviour with mesophyll demands for CO_2_ (von Caemmerer and Griffiths, 2009). The present study with starch-deficient RNAi lines of *K. fedtschenkoi* that displayed reduced nocturnal CO_2_ fixation supports the view that genetic manipulations that disrupt the diel cycle of the accumulation and turnover of malate and other primary metabolites have a profound influence on the temporal control and optimization of the CAM-associated rhythms of CO_2_ fixation and stomatal conductance (Dever *et al.*, 2015; Boxall *et al.*, 2017; Boxall *et al.*, 2020). Further work is required to determine whether and how accumulation of malate in the mesophyll and its transport to guard cells connects CAM photosynthesis in the mesophyll with stomatal behaviour over the diel cycle, whilst maintaining CO_2_ responsiveness over duration of the night.

### Starch deficiency curtails daytime stomatal closure

The substantial elevation in *C*_i_ that accompanies the decarboxylation of malate in the photoperiod is believed to be a key driver for stomatal closure in the light (Males and Griffiths, 2017). Curtailed daytime closure of stomata was noted in two starch-deficient lines (*rPGM1a* and *rPGM1c*), and it was hypothesized that this was a consequence of limited malate decarboxylation in these lines. To test this hypothesis, wild type and *rPGM1a* were exposed to CO_2_-free air overnight to abolish nocturnal malate accumulation, with the prediction that both genotypes would increase stomatal conductance the following day. The data showed that although wild-type plants increased stomatal conductance for the first few hours of the photoperiod after a night in CO_2_-free air, they still showed complete stomatal closure during phase III. The *rPGM1a* plants showed little change in daytime stomatal conductance after a night in CO_2_-free air. Thus, as surmised for stomatal behaviour at night, *C*_i_ is not the only factor influencing the daytime behaviour of CAM stomata (von Caemmerer and Griffiths, 2009). It is noteworthy in this context that Lefoulon *et al.* (2020) reported that guard cell anion channel activity in *K. fedtschenkoi* CAM leaves assayed in the light period tracked the temporal pattern of transcript abundance cycling of the genes encoding the channel proteins. Thus, the data presented here, taken together with those reported previously by von Caemmerer and Griffiths (2009) and Lefoulon *et al.* (2020), are consistent with the proposal that the circadian clock regulates the transcript oscillations of guard cell anion channel genes and through this mediates stomatal closure in the light period in *Kalanchoë* leaves regardless of metabolic or genetic interventions that reduce nocturnal CO_2_ fixation and vacuolar malate accumulation during the preceding dark period.

Starch deficiency was found to have profound impacts on the responsiveness of stomata to low and elevated [CO_2_] administered during the day. The wild-type stomata showed no (phase III) or limited (phase IV) stomatal opening in response to low [CO_2_], whereas the starch-deficient plants showed very marked stomatal opening, particularly in phase IV, when stomatal conductance values were >3.5 times higher than that measured for wild type. Moreover, although stomata in *rPGM1a* closed in response to elevated external [CO_2_] (i.e. 1600 µmol CO_2_ mol^−1^ air), stomata did not remain closed under this treatment. Together, the data indicate that starch biosynthesis is required for sustained daytime stomatal closure in *K. fedtschenkoi*. Substantial deposits of starch were found in the guard cells of *K. fedtschenkoi* and, as reported previously, this starch was not mobilized during the day as is the case in *Arabidopsis* (Abraham *et al.*, 2020). Such findings support the hypothesis of a crucial role for starch synthesis/accumulation in daytime stomatal closure in CAM.

A comparison of stomatal responses to [CO_2_] in mutants of *Arabidopsis* lacking starch in both mesophyll and guard cells with mutants lacking starch only in the mesophyll indicated that starch synthesis specifically in the guard cells was crucial for mediating [CO_2_]-induced stomatal closure (Azoulay-Shemer *et al.*, 2016). Guard cell starch synthesis is thought to play an essential role in CO_2_-induced stomatal closure by acting as a sink for C skeletons coming from malate degradation via gluconeogenesis during guard cell osmotic adjustment (Azoulay-Shemer *et al.*, 2016). Starch might also act as a sink for sugars produced via the Calvin cycle, and the disruption of starch synthesis increases sucrose content, which, together with its degradation products, fructose and glucose, is involved in stomatal osmoregulation (Lawson *et al.*, 2014; Azoulay-Shemer *et al.*, 2015). In the present study, the inability of *rPGM1a* to convert osmolytes such as malate and sucrose into insoluble starch granules in the guard cells in addition to the mesophyll could explain the incomplete daytime stomatal closure in these starch-deficient plants. The significantly higher soluble sugar content noted in guard cell-enriched epidermal peels and mesophyll from *rPGM1a* support the hypothesis that soluble sugars increased guard cell turgor pressure and curtailed complete closure of stomata during the day in the starch-deficient plants. Future testing of this hypothesis will require a more targeted genetic approach to manipulate starch metabolism in the CAM guard cell independently of that in the mesophyll and thereby establish the functional significance of the diel rescheduling of starch turnover in CAM guard cells, as reported here and previously (Abraham *et al.*, 2020).

### Future work and conclusions

The engineering of the inverted stomatal rhythm of CAM into non-CAM species offers the potential to increase WUE of important crops for human consumption and thus the productivity of arid environments. At night, CAM stomata are thought to open in response to reduced *C*_i_ caused by the consumption of CO_2_ by PEPC and the accumulation of malate (Males and Griffiths, 2017). According to Niechayev *et al.* (2019), in order to engineer the CAM stomatal rhythm successfully into non CAM, it is necessary that the host species have guard cells fully responsive to changes in [CO_2_] that allows the nocturnal stomatal opening and the diurnal closure. A first approach in the modification of *Arabidopsis* stomatal conductance has been made by Lim *et al.* (2019), who overexpressed genes implicated in both the carboxylation and decarboxylation modules of the facultative CAM species *M. crystallinum*. Transgenic lines overexpressing the carboxylation enzymes phosphoenolpyruvate carboxylase 1 (PEPC1), NAD^+^ malate dehydrogenase (NAD^+^ MDH), NADP malate dehydrogenase (NADP-MDH) and phosphoenolpyruvate carboxylase kinase (PPCK1), respectively, showed increased stomatal conductance. In contrast, lines overexpressing the decarboxylation module enzymes, such as NAD-malic enzyme 1 and 2 (NAD-ME1 and NAD-ME2) and NADP-malic enzyme (NADP-ME), showed decreased stomatal conductance and showed depletion of the content of organic acids.

Apart from the carboxylation and decarboxylation modules, engineering starch turnover in C_3_ guard cells with an increased content during the day followed by degradation at beginning of the night, as observed here and previously (Abraham *et al.*, 2020), will confirm whether the reprogramming of starch metabolism in guard cells is crucial for the inverted stomatal rhythm in CAM plants. In the same way, based on the different starch degradation pathways in the mesophyll that CAM and C_3_ follow (Ceusters *et al.*, 2021), a rerouting to the phosphorolytic pathway in C_3_ species will contribute to understanding how this pathway affects stomatal behaviour as a possible provider of substrates in the synthesis of nocturnal amino acids that can act as osmolytes in the guard cells. To achieve this, it is also necessary to elucidate whether differences in both guard cells and mesophyll starch turnover in CAM are under circadian regulation and whether this altered clock control has to be engineered into C_3_ plants to allow the inverted stomatal rhythm.

In conclusion, we have shown that the nocturnal opening of stomata in the constitutive CAM species *K. fedtschenkoi* is not reliant upon nocturnal starch degradation, as evidenced by comparable stomatal conductance and opening in response to low [CO_2_] administered at night in wild-type and starch-deficient plants of *K. fedtschenkoi*. Starch synthesis was required for stomatal closure in the light, potentially acting as a sink for soluble sugars and/or malate, thereby promoting a reduction in guard cell turgor, as illustrated in our proposed model (Supplementary data Fig. S3). This hypothesis was supported by marked accumulation of soluble sugars in both the mesophyll and guard cell-enriched epidermis of *rPGM1a* during the day. Data also implicate the importance of malate accumulation in the mesophyll and its transport into guard cells, which appears necessary for driving nocturnal stomatal opening and for connecting CAM photosynthesis in the mesophyll with stomatal behaviour. Together, the findings reported here indicate that the nocturnal degradation and daytime synthesis of starch in this CAM species regulates sugar and malate homeostasis between mesophyll and guard cells and within the guard cells per se. These processes, in turn, have profound implications for the diel control of stomatal conductance and responsiveness to [CO_2_] across the diel CAM cycle.

## SUPPLEMENTARY DATA

Supplementary data are available online at https://academic.oup.com/aob and consist of the following. Figure S1: stomatal impressions from wild type and *rPGM1a* for anatomical measurements of stomatal size and density during 24 h. Figure S2: relative transcript abundance of Kf*-PGM*, Kf*-ABCB14*, Kf*-STP1* and Kf*-SUSY1* genes in wild type and *rPGM1a* in mesophyll and guard cell-enriched epidermis at dawn and dusk. Figure S3: proposed model of impaired stomatal closure during the day period in *rPGM1a* plants of *Kalanchoë fedtschenkoi*.

mcad017_suppl_Supplementary_Figure_S1

mcad017_suppl_Supplementary_Figure_S2

mcad017_suppl_Supplementary_Figure_S3

mcad017_suppl_Supplementary_Data
