# Supplementary figures and images for: The starch-deficient plastidic PHOSPHOGLUCOMUTASE mutant of the constitutive crassulacean acid metabolism (CAM) species Kalanchoë fedtschenkoi impacts diel regulation and timing of stomatal CO2 responsiveness
Source: Ann Bot. 2023 Jan 20;132(4):881–94. doi: 10.1093/aob/mcad017 (PMC10799981; doi:10.1093/aob/mcad017)

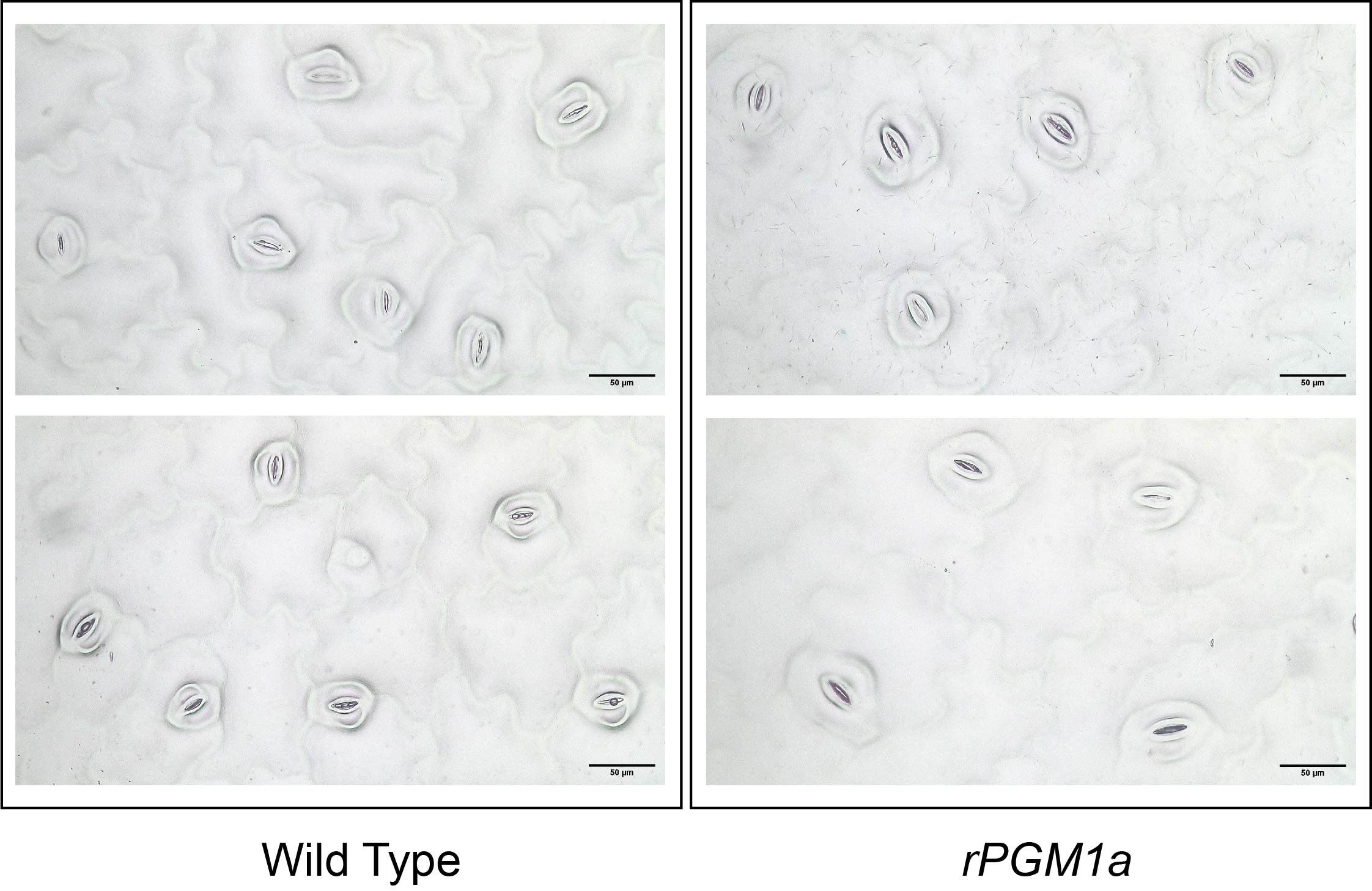

Supplement: mcad017_suppl_Supplementary_Figure_S1 [file mcad017_suppl_supplementary_figure_s1.jpeg]

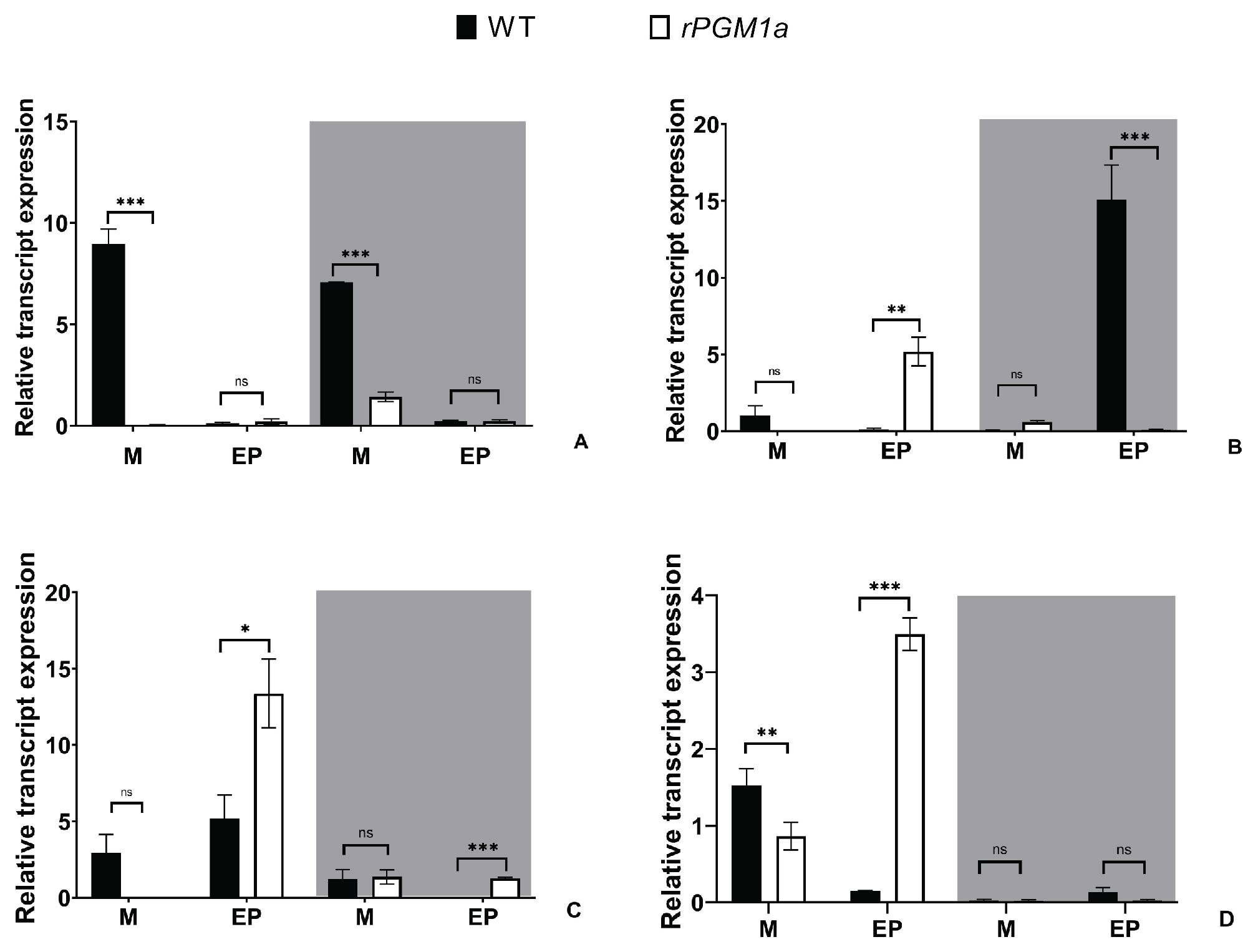

Supplement: mcad017_suppl_Supplementary_Figure_S2 [file mcad017_suppl_supplementary_figure_s2.jpeg]

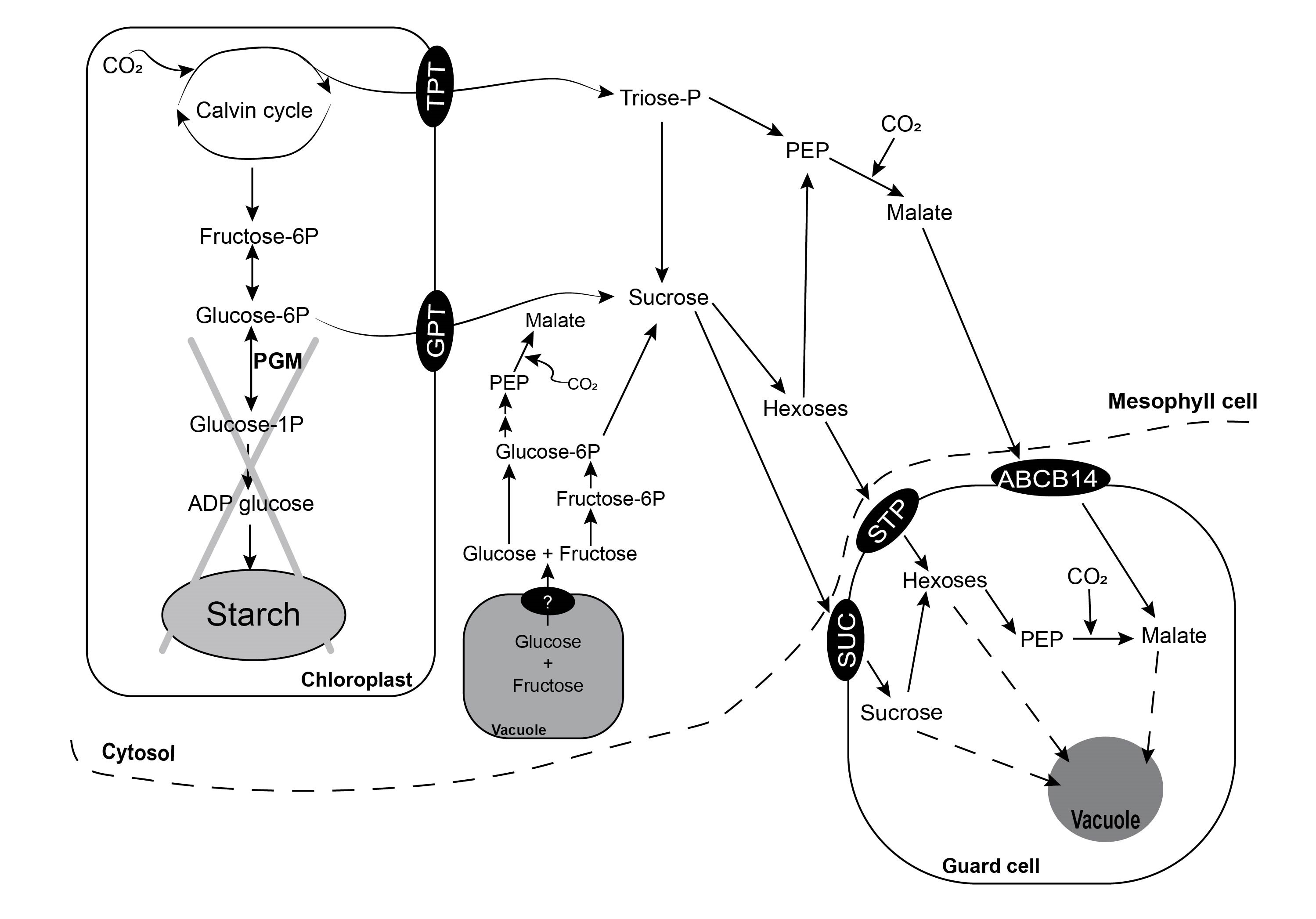

Supplement: mcad017_suppl_Supplementary_Figure_S3 [file mcad017_suppl_supplementary_figure_s3.jpeg]
