# Supplementary material for: The starch-deficient plastidic PHOSPHOGLUCOMUTASE mutant of the constitutive crassulacean acid metabolism (CAM) species Kalanchoë fedtschenkoi impacts diel regulation and timing of stomatal CO2 responsiveness
Source: Ann Bot. 2023 Jan 20;132(4):881–94. doi: 10.1093/aob/mcad017 (PMC10799981; doi:10.1093/aob/mcad017)
Supplement: mcad017_suppl_Supplementary_Data [file mcad017_suppl_supplementary_data.docx]

**SUPPLEMENTARY DATA CAPTIONS**

**Figure S1.** Stomatal impressions from wild type and *rPGM1a* for anatomical measurements of stomatal size and density during 24 hours. The images corresponded to upper (up) and lower (low) epidermal surfaces.

**Figure S2.** Relative transcript abundance of Kf*-PGM* (A), Kf*-ABCB14* (B), Kf*-STP1* (C) and Kf*-SUSY1* (D) genes in wild type (black) and *rPGM1a* (white) in mesophyll (M) and guard cell-enriched epidermis (EP) at dawn and dusk. Leaf pair 6 was used for this analysis. The error bars represent the standard error of six replicates (3 biological replicates, each with 2 technical replicates). Asterisks (*) indicate significant statistical difference between genotypes determined by one-way ANOVA (*p*< 0.05).

**Figure S3.** Proposed model of impaired stomatal closure during the day period in *rPGM1a* plants of *Kalanchoë fedtschenkoi*. The results obtained suggest that starch synthesis acts as a sink for sugars, promoting diurnal stomatal closure, demonstrated by the higher accumulation of soluble sugars in the *rPGM1a* during the day. X depicts silencing of phosphoglucomutase enzyme (PGM) and lack of starch synthesis. Transporters shown within black circles are; triose-P translocator (TPT), glucose-6-P translocator (GPT), sucrose transporter (SUC), sugar transporter protein (STP) and ATP-binding cassette transporter 14 (ABCB14).
